# Tastes of sustainability: a regional journey through Chilean traditional diets

**DOI:** 10.1590/0102-311XEN264725

**Published:** 2026-08-31

**Authors:** Rebecca Kanter, Mariana León V., Viviana Azúa Aros, Karla Jordan Manríquez

**Affiliations:** 1 Universidad de Chile, Santiago, Chile.; 2 Universidad Central de Chile, Santiago, Chile.

**Keywords:** Healthy Diet, Dietary Habits, Plant-based Diet, Environmental Sustainability, Dieta Saludable, Hábitos Alimentarios, Dieta a Base de Plantas, Sostenibilidad Ambiental, Dieta Saudável, Hábitos Alimentares, Dieta Baseada em Plantas, Sustentabilidade Ambiental

## Abstract

Traditional diets are generally associated with health benefits and are commonly recommended in food-based dietary guidelines. As climate change intensifies in the 21st century, food-based dietary guidelines have begun integrating environmental sustainability considerations. Despite growing global efforts to incorporate sustainability into food-based dietary guidelines, few studies have explored how to operationalize such guidance. A multiregional participatory study was conducted with adults from the central urban metropolitan area and the La Araucanía Region, in southern Chile, using semistructured interviews combined with card-sorting activities. The most commonly consumed and preferred traditional culinary preparations from each region were presented on cards and sorted according to participants’ perceptions across five dimensions of sustainable diets. In both regions, over 70% of participants reported physical access to most traditional culinary preparations, although economic access varied. In the Metropolitan Area, perceptions of environmental impact were more diverse, while participants in the La Araucanía Region generally viewed traditional foods as sustainable due to their natural origins. Taste preference was high across preparations; however, only fruits and salads were consumed almost daily. Only six traditional preparations scored below 60% for overall sustainability. These findings highlight important regional differences and gaps between taste preferences, perceptions of environmental sustainability, and actual consumption of sustainable traditional culinary preparations. They also demonstrate the value of participatory research in identifying sustainable and healthy food options that can support food-based dietary guidelines in territorially diverse populations.

## Abstract (es)

En general, las dietas tradicionales están asociadas a beneficios para la salud y ser ecomiendan en las guías alimentarias. Ante el empeoramiento del cambio climático en el siglo XXI, las guías alimentarias empezaron a integrar la sostenibilidad ambiental. A pesar de los crecientes esfuerzos globales por incorporar la sostenibilidad en las guías alimentarias, aún existen pocos estudios sobre cómo implementar dichas directrices. Se realizó un estudio participativo multirregional con adultos de la zona urbana central de la Región Metropolitana y de la Región de La Araucanía, en el sur de Chile, mediante entrevistas semiestructuradas que incluían actividades de clasificación de tarjetas. Se presentaron en tarjetas las preparaciones culinarias tradicionales más consumidas y apreciadas en cada región para que los participantes las clasificaran según sus percepciones sobre cinco dimensiones de las dietas sostenibles. Aunque la asequibilidad variaba, más del 70% de los participantes tenían acceso físico a la mayoría de las preparaciones culinarias tradicionales en ambas regiones. En la Región Metropolitana, existía una mayor diversidad en las percepciones sobre el impacto ambiental, mientras que en la Región de La Araucanía, los alimentos tradicionales casi siempre se consideraban sostenibles debido a su origen natural. La preferencia por el sabor fue alta entre las preparaciones, pero solamente las frutas y las ensaladas se consumían casi a diario. Solo seis preparaciones tradicionales obtuvieron una puntuación inferior al 60% en sostenibilidad general. Estos hallazgos resaltan importantes diferencias regionales y brechas entre la preferencia por el sabor, los diversos valores ambientales y el consumo alimentario real de preparaciones culinarias tradicionales sostenibles. Esto demuestra el valor de la investigación participativa para identificar opciones alimentarias saludables y sostenibles que respalden las guías alimentarias para poblaciones territorialmente diversas.

## Abstract (pt)

Em geral, dietas tradicionais estão associadas a benefícios à saúde e são recomendadas nos guias alimentares. Diante do agravamento das mudanças climáticas no século XXI, os guias alimentares começaram a integrar a sustentabilidade ambiental. Apesar dos esforços globais crescentes para incorporar a sustentabilidade nos guias alimentares, poucos estudos existem sobre como operacionalizar tais orientações. Foi realizado estudo participativo multiregional com adultos da Região Metropolitana central urbana e da Região de La Araucanía, no sul do Chile, através de entrevistas semiestruturadas com atividades de seleção de cartões. Preparações culinárias tradicionais mais consumidas e apreciadas em cada região foram colocadas em cartões que os participantes classificaram com base em percepções sobre cinco dimensões de dietas sustentáveis. Em ambas as regiões, mais de 70% dos participantes tinham acesso físico à maioria das preparações culinárias tradicionais, embora o acesso econômico variasse. Na Região Metropolitana, havia mais diversidade nas percepções sobre o impacto ambiental, enquanto na Região da La Araucanía, os alimentos tradicionais eram quase sempre vistos como sustentáveis devido à sua origem natural. A preferência pelo sabor foi alta entre as preparações, mas apenas frutas e saladas eram consumidas quase diariamente. Apenas seis preparações tradicionais tiveram pontuação abaixo de 60% em sustentabilidade geral. Esses achados destacam diferenças regionais importantes e lacunas entre preferência de sabor, diversos valores ambientais e consumo alimentar real de preparações culinárias tradicionais sustentáveis. Isso demonstra o valor da pesquisa participativa na identificação de opções alimentares saudáveis sustentáveis para apoiar os guias alimentares para populações territorialmente diversas.

## Introduction

Within the global nutrition transition, Chile is a global leader in both ultra-processed food and beverage sales and obesity rates ^1^. In addition to harming human health, ultra-processed foods lead to greater environmental degradation ^2^. The concomitant increase in ultra-processed food and beverage consumption and climate change has contributed to a global syndemic of climate change, undernutrition, and obesity, signaling an urgent need to increase consumption of sustainable diets ^3^ ^,^ ^4^ ^,^ ^5^. Concepts related to environmental sustainability are being increasingly included in food-based dietary guidelines, most recently in Chile and Mexico ^6^ ^,^ ^7^ ^,^ ^8^. However, such food-based dietary guidelines often do not address broader concepts of sustainable diets, let alone provide related guidance. Sustainable diets, defined by the Food and Agriculture Organization of the United Nations (FAO) and Bioversity International, are not limited to environmental impacts but encompass five dimensions, including cultural acceptability, physical accessibility, economic accessibility, and nutritional adequacy ^9^. Few studies have investigated the operationalization of sustainable diets as a means to meet food-based dietary guidelines ^10^ ^,^ ^11^ ^,^ ^12^ ^,^ ^13^. These studies have largely focused on the environmental dimension of sustainable diets and the related environmental assessment of current dietary practices. This confluence of food and nutrition crises with increasing environmental degradation underscores an urgent need for novel analyses and new paradigms to characterize sustainable diets.

Chile, one of the longest countries in the world, is characterized by a wide range of microclimates. As an agro-export power with a rich history of local food systems, exemplified by outdoor markets (*ferias libres*) that have existed since the early 20th century, Chile provides a compelling context for studying sustainable diets ^14^. Furthermore, the country faces substantial human and planetary health challenges, as reflected in the high prevalence of overweight and obesity and the increasing frequency of extreme weather events due to climate change ^15^ ^,^ ^16^. In Chile, early 20th century diets and recipe books that are widely used by the general population reflect local resources and cultural contexts through their ingredients and culinary preparations ^17^ ^,^ ^18^. Traditional diets worldwide are associated with positive health outcomes, and their incorporation into contemporary food systems may contribute to healthier and sustainable diets ^19^ ^,^ ^20^. The new Chilean food-based dietary guidelines aim to improve both population and environmental health via ten concrete messages based on six overarching principles, including adopting sustainable diets, strengthening sustainable food systems, prioritizing minimally processed foods, protecting Chile’s biodiversity, and valuing food cultures, especially by means of homemade foods ^7^. However, the Chilean food-based dietary guidelines do not mention healthy foods that characterized the traditional Chilean diet of previous generations ^7^ ^,^ ^18^. Food is also recognized as a cultural expression rooted in place ^21^. Thus, eating transcends mere nutrition and becomes a profoundly social and cultural practice. From a socioanthropological perspective, eating is a key dimension in the construction of individual and collective identities ^22^ ^,^ ^23^. To address this gap, we sought to better understand traditional food consumption in Chile and how it can be used to promote healthy and sustainable diets.

Geographically distinct regions in Chile, as elsewhere, differ in their territorial realities and cultural contexts. Understanding these differences is crucial to support the adoption of more sustainable diets at the population level. Compared to previous dietary guidelines, the new Chilean food-based dietary guidelines acknowledge these diverse territorial and cultural realities while recognizing the need to make food systems more sustainable ^7^. However, like other food-based dietary guidelines that promote traditional culinary preparations, the Chilean food-based dietary guidelines do not address how recommendations (e.g., eat a colorful diet) can be appropriately adapted to ensure sustainable diets in different contexts ^24^ ^,^ ^25^. We previously quantified the impacts of five dimensions of sustainable diets for traditional culinary preparations in the Metropolitan Area, including their environmental footprint ^26^. However, quantitative assessments of sustainability-related impacts may overlook household-level realities, making it difficult to implement dietary guidance in practice. The primary and secondary objectives of this study were to: (i) assess the sustainability of selected traditional Chilean culinary preparations across five dimensions of sustainable diets (cultural acceptability, environmental protection, physical accessibility, economic accessibility, and nutritional adequacy); and (ii) explore how these preparations may support the promotion of sustainable diets in two regions of Chile (the Metropolitan Area and the La Araucanía Region). The study reported here is part of the *Healthy and Sustainable Diets for Chileans: Recovering Traditional Diets* project ^27^, a participatory mixed-methods study to investigate and promote sustainable diets. The adaptation of sustainable diets will help improve both human and planetary health.

## Materials and methods

### Study area

The study was conducted in Chile’s central Metropolitan Area and southern La Araucanía Region, which are separated by 617 kilometers (383 miles). The Metropolitan Region, an urban area, is home to over half of Chile’s population, while the La Araucanía Region is one of the most rural regions of Chile’s 16 regions ^28^. Participatory research activities were conducted in almost half of all counties in the Metropolitan Area (n = 25/52 counties) and 43% of counties in the La Araucanía Region (n = 14/32 counties). Counties were selected based on socioeconomic, sociocultural (e.g., prevalence of Indigenous communities), and territorial diversity (urban/rural settings and geographical diversity, such as valleys). More details about the study site selection have been previously described ^27^.

### Study participants

Participation was limited to individuals who met the inclusion criteria of self-declared knowledge about the preparation of traditional Chilean recipes and being 25 years or older. We applied the same sampling approach in both regions, with 40 individuals purposively selected per region via word-of-mouth recruitment and public advertisements. This sample size was considered sufficient to achieve thematic and perceptual saturation ^29^. Specifically, participants were recruited to ensure representation across age groups (25-45 years, 46-64 years, ≥ 65 years) and ethnic backgrounds (i.e., Indigenous and non-Indigenous participants), with approximately eight participants in each subgroup. Participants were informed that the project focused on traditional Chilean foods, and written informed consent was obtained prior to data collection. Three trained interviewers conducted the interviews at times and locations chosen by the participants. Interviews were most often conducted in participants’ homes, but also included libraries, cafes, and outdoor plazas. All interviews were audio-recorded and transcribed verbatim.

### Methods

The study *Healthy and Sustainable Diets for Chileans: Recovering Traditional Diets* aimed to generate critical insights about the sustainability of traditional Chilean diets and to identify culinary preparations for healthy diet interventions and future policies and programs. The research presented here is a subcomponent of this larger study. Participant recruitment and data collection for the present analysis was conducted between April 18, 2018 and September 3, 2019.

Each participant completed a 90-minute semi-structured individual interview. The interviews involved activities about 24 traditional culinary preparations in the Metropolitan Area and 27 in the La Araucanía Region. The interview combined card-sorting activities with semi-structured interviewing to explore participants’ perceptions of sustainable diets of traditional Chilean culinary preparations and to better understand the meanings underlying those perceptions within specific contexts. A semi-structured interview guide and a card-sort protocol were developed for this purpose. The activities were the same across regions and included card-sorting exercises based on sustainable diet criteria, as well as two brief surveys assessing diet and taste preferences for each culinary preparation. Card sorting is a method commonly used in cultural domain analysis, in which participants organize cards into groups according to their perceptions of a given topic ^30^. Two sets of cards depicting traditional culinary preparations were developed: 24 for the Metropolitan Area and 27 for the La Araucanía Region. These preparations were selected based on previous focus groups and represented those that were both commonly consumed and highly preferred in terms of taste. Further details regarding the selection process have been described elsewhere ^27^. Participants were tasked with sorting or grouping cards, each displaying an image of a different traditional culinary preparation, to identify and characterize perceptions related to sustainable diets.

During the card-sorting interview, participants were asked to sort the culinary preparation cards multiple times (n = 2-7) across five different contexts: (1) menus representing what they would consume during on a typical day; (2) environmental impact (positive or negative); (3) physical accessibility based on where they live and/or work (if applicable); (4) economic feasibility; and (5) nutritious menus or groups of culinary preparations. Participants were also asked to identify any foods or beverages they considered important but that were not represented among the culinary preparation cards. If participants did not know how to sort certain cards (i.e., culinary preparations), they were assured that they could leave them out of the card sort piles.

In both regions, a food frequency questionnaire and a taste preference questionnaire were administered after the card-sorting activity to assess current consumption patterns and taste preferences for each culinary preparation included in the card sort. The food frequency questionnaire was semi-quantitative and analyzed both the frequency of consumption (i.e., daily, more than once a month, once a week, on special occasions, or never) and the usual portion size consumed via a self-reported open-ended question. For the taste preference questionnaire, participants were asked to rate their preference for each culinary preparation using a five-point Likert scale represented by smiley faces, ranging from 1 (“do not like at all!”), 2 (“do not like it”), 3 (“I like it okay”), 4 (“I like it”), to 5 (“love it!”). There questionnaire also included space for interviewers to record any additional comments related to participants’ taste preferences for specific culinary preparations. All interviews were recorded and transcribed. The study design is described in detail in Kanter & León Villagra ^27^.

Data collected from the card-sorting forms, food frequency questionnaires, and taste preference questionnaires were entered into the Research Electronic Data Capture (REDCap; https://project-redcap.org/), a secure web-based application for building and managing surveys and databases ^31^.

### Ethics statement

All participants provided written informed consent prior to their participation in the study. Data collection was approved by the Ethics Committee for Human Subjects Research of the University of Chile’s Faculty of Medicine (application number 164-2017).

### Card-sorting activity

#### Definition of variables

First, participants were shown all the cards corresponding to their region and asked to create one or more daily menus representing what an average member of their household might eat. They could use any number of cards, repeat cards across menus, and create as many menus (i.e., card piles) as they wished. After completing each menu, they described it in their own words, explaining how they imagined consuming the dishes throughout the day (e.g., a typical weekend menu, a special-occasion menu, or a summer menu). During this activity, participants from both regions often requested or used additional non-photographic cards that had been added by earlier participants (e.g., bread, butter, water, and potato casserole). At the end of this first exercise, participants also reported their usual weekday and weekend meal times (e.g., breakfast, lunch, and dinner). Second, participants sorted the cards into two piles according to whether they believed each culinary preparation had a positive or negative environmental impact. No examples were provided to avoid any potential bias. After sorting, they explained the reasoning behind their choices. Third, participants sorted the cards based on physical accessibility. They could assess accessibility in terms of either the prepared dish (e.g., availability of a vegetable casserole at restaurants) or the main ingredients required to prepare it. Some participants created four piles instead, distinguishing between greater versus lower access to ingredients and greater versus lower access to prepared dishes. Fourth, participants sorted the cards into two piles according to whether they perceived each culinary preparation (or its main ingredients) as economically accessible. Finally, participants sorted the cards based on perceived nutritional adequacy, identifying which preparations they considered nutritionally adequate and creating a menu they perceived as nutritionally adequate.

#### Participant characteristics

All collected sociodemographic characteristics were used to describe the study population, including age (years), gender (man, woman, other), ethnicity, and occupation. Both ethnicity and occupation were collected via open-ended questions and subsequently converted into categorial variables. Ethnicity was categorized according to self-identified Indigenous affiliation (e.g., Mapuche) or non-Indigenous status. Occupation was categorized into the following sectors: homemaker, education, retired, healthcare, business, food service, agriculture, and other.

#### Semi-quantitative food frequency questionnaire

For each region, the frequency of consumption of each culinary preparation was recorded using categories ranging from never to every day. Portion sizes were not standardized and were based on participants’ self-reported estimates (e.g., one small plate, one plate, one large plate, half a cup, or two tablespoons). However, many portion-size estimates were repeated across participants. In the La Araucanía Region, where food culture differs markedly compared to the Metropolitan Area, culinary preparations were also categorized as seasonal when applicable.

#### Taste preference score

Taste preference was assessed using a five-point Likert scale represented by facial expressions, with response options ranging from 1 (“do not like at all!”), 2 (“do not like it”), 3 (“I like it okay”), 4 (“I like it”), to 5 (“love it!”).

### Data analysis

Sociodemographic data collected during the card sort activity were used to create a summary table of participant characteristics. Mean age and distribution of participants by gender, ethnicity, and occupation were calculated separately for each region. The chi-squared test was performed to assess statistically significant differences (p < 0.05) between regions for each sociodemographic variable.

Food frequency categories were converted into estimated weekly consumption frequencies (i.e., number of times consumed per week). Mean taste preference scores were calculated for each traditional culinary preparation included in the card-sorting activity.

The analysis of the card-sorting activity was conducted separately for each region and involved several steps. For each culinary preparation, the total number of participants who classified the preparation positively for each of the five sustainability criteria (e.g., positive environmental impact) was calculated and the proportion out of 40 participants was determined. The corresponding total number and proportion of participants that classified each culinary preparation as negative was also determined. These results were summarized into a table in which physical availability was listed as the first dimension. This ordering reflects the premise that, if a culinary preparation is not physically possible, it is not feasible to implement the subsequent dimensions of economic accessibility, environmental impact, and perceived nutritional adequacy (i.e., “healthfulness”). The cultural acceptability dimension was not included in this analysis because all participants utilized all cards during the activity, implying universal cultural acceptability.

#### Sustainability score by region

For each culinary preparation, an average sustainability score (0-100%) was calculated based on participants’ positive classifications across the five dimensions of sustainable diets. Each dimension was assigned equal weight (1/5). For example, a culinary preparation received a positive classification for a given participant if it was: (1) included in menus that the participant would typically consume; (2) perceived as having positive environmental impact; (3) considered physically accessible; (4) considered economically accessible; and (5) perceived as nutritionally adequate. For each culinary preparation, the mean sustainability score was calculated across all participants in each region. Culinary preparations were subsequently ranked in descending order and categorized as having high (75-100%), medium (60-74%), or low (< 60%) perceived overall sustainability. A high sustainability rating indicated that most participants positively associated the culinary preparation with four or five sustainability dimensions. A medium rating indicated that most participants positively associated the preparation with approximately three or four dimensions. A low rating indicated that participants positively associated the preparation with three or fewer dimensions. Additionally, for each culinary preparation, the proportion of participants who identified it as contributing positively to each dimension was calculated. For example, 24 of 40 individuals (60%) in the Metropolitan Area identified the card “tea/coffee/mate” as positively impacting the environment. These proportions were also categorized as high (75-100%), medium (50-74%) or low (< 50%).

#### Qualitative data analysis

Trained professionals transcribed the interviews that were then uploaded into the ATLAS.ti qualitative software (http://atlasti.com/). Qualitative data analyses were performed using ATLAS.ti v. 8.3.1. To complement and further elucidate the quantitative card-sorting results, specific culinary preparations were selected for in-depth qualitative analysis. The culinary preparations were chosen according to criteria shared across both regions: a preparation identified as highly sustainable, liked, and consumed (eggs); preparations identified as sustainable and highly liked but less consumed (legumes and cazuelas); and preparations that were highly liked but neither widely perceived as sustainable nor highly consumed (fish/seafood soup and ulpo in the Metropolitan Area, and piñones in the La Araucanía Region). For these six culinary preparations, research assistants trained in qualitative methods coded interview transcripts based on four dimensions of sustainable diets: physical accessibility, economic accessibility, environmental impact, and nutritional adequacy. The resulting codes were then grouped into broader themes to identify perspectives explaining why these culinary preparations aligned with the previously described criteria.

## Results

The 80 participants included 17 men, 62 women, and one gender-fluid individual, ranging in age from 25 to 86 years. Overall, 27% identified as Indigenous (Table 1). In both regions, homemakers were the largest occupational group, followed by participants working in education in the Metropolitan Area (20%) and in business in the La Araucanía Region (12%). No significant sociodemographic differences were found between regions. Across both regions, 80% or more of participants reported physical accessibility to most culinary preparations (Table 2). Perceptions of economic accessibility showed greater variability in the Metropolitan Area, where only nine preparations were widely considered economically accessible, compared with 14 in the La Araucanía Region. In both regions, salads, eggs, and tea/coffee were consistently viewed as economically accessible. Environmental impact perceptions also varied more in the Metropolitan Area. Of the 24 dishes assessed, nine were widely perceived as environmentally positive (e.g., seaweed salad, fruit, and vegetable soup), nine received moderate ratings (e.g., legumes and chicken), and six were perceived positively by only a minority of participants (e.g., avocado, shredded beef, and seafood). In contrast, nearly all dishes in the La Araucanía Region were perceived as environmentally positive; only three received moderate ratings: chicken with peas (73%), cazuelas (70%), and mussel/seafood soup (68%). In both regions, about half of the dishes were perceived as nutritionally adequate, particularly fruit, legumes, and seaweed (*cochayuyo*) salad. However, perceptions of the least nutritious dishes differed between regions. In the Metropolitan Area, these included “leftovers for lunch” and empanadas, whereas in the La Araucanía Region, they included homemade jam and kuchen (*kugen*) cake.

**Table 1:** Study sample characteristics.

	Metropolitan	La Araucanía	Total
Total [n (%)]	40 (50)	40 (50)	80 (100)
Age [years (SD)]	52 (15)	50 (17)	51 (16)
Gender [n (%)]			
Woman	30 (75)	32 (80)	62 (78)
Man	10 (25)	7 (18)	17 (21)
Gender fluid	0 (0)	1 (2)	1 (1)
Ethnicity [n (%)]			
Non-Indigenous	30 (75)	28 (70)	58 (72)
Indigenous (Mapuche)	8 (20)	12 (30)	20 (25)
Indigenous (Aymara)	2 (5)	0 (0)	2 (2)
Occupation [n (%)]			
Homemaker	9 (22)	14 (35)	23 (29)
Education	8 (20)	3 (8)	11 (14)
Art	7 (18)	1 (2)	8 (10)
Retired	3 (8)	5 (12)	8 (10)
Health	3 (8)	3 (8)	6 (8)
business	2 (5)	5 (12)	7 (9)
Restaurant industry	1 (2)	3 (8)	4 (5)
Agriculture	1 (2)	2 (5)	3 (4)
Other	6 (15)	4 (10)	10 (12)

**Table 2:** Regional perceptions of sustainable diet dimensions by culinary preparation.

Culinary preparation	Physical accessibility	Economic availability	Positive environmental impact	Nutritionally positive
n (%)	n (%)	n (%)	n (%)
Metropolitan Region				
Eggs with tomato or onion	39 (98)	36 (90)	20 (50)	27 (68)
Salads: Chilean salad, or tomato, or mixed salad	38 (95)	31 (78)	31 (78)	33 (83)
*Charquican*: a warm dish with ground beef, potatoes, pumpkin, white corn, onion, peas and corn; and often topped with a fried egg	38 (95)	27 (68)	23 (58)	33 (83)
Fruit: plain or fruit salad	37 (93)	31 (78)	33 (83)	38 (95)
Vegetable soup	37 (93)	35 (88)	32 (80)	29 (73)
Legumes: lentils or beans or *garbanzos*	36 (90)	26 (65)	31 (78)	38 (95)
Vegetable casserole or vegetable stew	36 (90)	36 (90)	32 (80)	23 (58)
Tea/Coffee/Mate	35 (88)	34 (85)	24 (60)	26 (65)
*Porotos con riendas*: boiled cranberry beans with spaghetti, sausage, diced pumpkin, dried chili condiment, and onions	35 (88)	25 (63)	19 (48)	32 (80)
*Tortilla* de verduras: eggs with vegetables (*frittata* style)	35 (88)	35 (88)	30 (75)	30 (75)
*Causeo*: tomato mixed with tuna fish	35 (88)	29 (73)	21 (53)	28 (70)
Shredded beef	35 (88)	2 (5)	17 (43)	24 (60)
Whole wheat bread with avocado or avocado with onion	34 (85)	6 (15)	29 (73)	33 (83)
*Cazuelas*: boiled beef or chicken with chopped onions and carrot; and then added to the shallow serving dish is a piece of squash, potato, and sweet corn	34 (85)	13 (33)	18 (45)	36 (90)
Cheese with tomato	33 (83)	18 (45)	27 (68)	31 (78)
*Pantrucas*: Chilean dumpling stew	33 (83)	20 (50)	22 (55)	26 (65)
*Porotos granados* or *porotos con mazamorra*: a traditional Chilean stew of mainly cranberry beans, corn and squash	32 (80)	24 (60)	30 (75)	37 (93)
*Empanadas*: any kind	32 (80)	11 (28)	10 (25)	6 (15)
Baked chicken	32 (80)	24 (60)	16 (40)	24 (60)
Leftovers for lunch	30 (75)	24 (60)	24 (60)	19 (48)
Fish: fish ceviche or baked fish	28 (70)	3 (8)	18 (45)	36 (90)
*Cochayuyo* (seaweed salad)	28 (70)	30 (75)	34 (85)	38 (95)
Fish soup	27 (68)	8 (20)	24 (60)	37 (93)
*Ulpo*: toasted wheat flour with or without milk	25 (63)	32 (80)	28 (70)	21 (53)
La Araucanía Region				
Tea/Coffee	40 (100)	38 (95)	30 (75)	26 (65)
Scrambled eggs	40 (100)	40 (100)	31 (78)	38 (95)
*Cazuelas:* boiled beef or chicken with chopped onions and carrot; and then added to the shallow serving dish is a piece of squash, potato, and sweet corn	40 (100)	31 (78)	28 (70)	37 (93)
Mate	39 (98)	28 (70)	33 (83)	20 (50)
Salads	39 (98)	32 (80)	34 (85)	40 (100)
*Porotos con riendas:* boiled cranberry beans with spaghetti, sausage, diced pumpkin, dried chili condiment, and onions	39 (98)	34 (85)	33 (83)	37 (93)
Chilean salad: tomatoes, onions, cilantro	39 (98)	34 (85)	36 (90)	34 (85)
Legumes: lentils	39 (98)	37 (93)	39 (98)	39 (98)
*Carbonada*: beef stew	39 (98)	34 (85)	30 (75)	37 (93)
*Pollo arvejado:* chicken with peas	39 (98)	28 (70)	29 (73)	37 (93)
Homemade bread/*Tortilla al rescoldo*	38 (95)	37 (93)	34 (85)	27 (68)
*Kuchen*: pie-like pastry, with a thick, “cakey” crust and a sweet custard based or chopped fruit topping	38 (95)	23 (58)	33 (83)	12 (30)
Fruit	37 (93)	28 (70)	32 (80)	40 (100)
Legumes: *garbanzos* and/or peas	36 (90)	34 (85)	38 (95)	40 (100)
Leftovers for lunch	36 (90)	32 (80)	33 (83)	29 (73)
*Pantrucas*: Chilean dumpling stew	36 (90)	39 (98)	33 (83)	20 (50)
Avocado	35 (88)	5 (13)	31 (78)	40 (100)
Cheese	35 (88)	8 (20)	32 (80)	33 (83)
Cooked fava beans	34 (85)	27 (68)	38 (95)	37 (93)
Cooked sweetcorn	34 (85)	34 (85)	35 (88)	28 (70)
*Humitas*: mashed fresh sweet corn, onion, basil, and butter or lard wrapped in corn husks and baked or boiled	33 (83)	19 (48)	35 (88)	30 (75)
*Pastel de choclo:* a sweetcorn pudding in a shallow dish in which minced beef cooked with onions, paprika, other spices, and sometimes chicken is placed in the bottom of the dish with slices of hardboiled egg, olives and raisins	32 (80)	17 (43)	31 (78)	31 (78)
Homemade marmalade	32 (80)	20 (50)	34 (85)	14 (35)
*Cochayuyo* (seaweed) salad or with potatoes	27 (68)	32 (80)	34 (85)	40 (100)
Seafood or mussel soup	24 (60)	22 (55)	27 (68)	31 (78)
*Piñones:* edible pine seeds from the Chilean tree species *Araucaria araucana*	24 (60)	8 (20)	30 (75)	29 (73)
*Diguenes* (*Cyttaria espinosae*) salad: an orange-white colored edible fungus typically consumed as a salad	23 (58)	13 (33)	33 (83)	29 (73)

### Diet and taste preferences of traditional culinary preparations

In both regions, participants reported high taste preference scores for almost all culinary preparations (Table 3). Fruit and salads were universally liked. In the Metropolitan Area, whole-wheat bread with avocado and the traditional bean-and-corn stew *porotos granados* were also especially favored. In the La Araucanía Region, participants similarly expressed strong preferences for *cazuela*, pastel de *choclo*, and local foods such as *diguenes* (an edible fungus) and *piñones* (pine seeds). Despite these strong taste preferences, reported consumption varied. In both regions, fruits and salads were consumed nearly every day (Table 3). In contrast, *porotos granados* in the Metropolitan Area and pastel de *choclo* and *piñones* in the La Araucanía Region were consumed less than once per week. More broadly, several highly liked dishes, such as legumes in the La Araucanía Region, were consumed infrequently. Participants in the La Araucanía Region noted that legumes have become more expensive because many available varieties are now imported, despite the region’s history as a major national producer. As one participant explained, “*legumes are not expensive, legumes are expensive if you do not grow it yourself*”. Although several participants in the La Araucanía Region reported growing legumes during the growing season, average legume consumption in the region was less than once per week. In both regions, fish/seafood soup and the Chilean dumpling soup, *pantrucas*, were the least frequently consumed preparations (Table 3).

**Table 3:** Taste and diet preferences of the traditional dishes.

Culinary preparation	Average taste score (1-5)	Average weekly consumption (times per week)
Metropolitan Region		
Fruit: plain or fruit salad	5	5.7
Salads: Chilean salad, or tomato, or mixed salad	5	6.6
Whole wheat bread with avocado or avocado with onion	5	3.3
*Porotos granados* or *porotos con mazamorra*: a traditional Chilean stew of mainly cranberry beans, corn and squash	5	1.1
Tea/Coffee/Mate	4	6.9
*Ulpo*: toasted wheat flour with or without milk	4	0.2
Eggs with tomato or onion	4	1.8
Cheese with tomato	4	2.4
*Causeo*: tomato mixed with tuna fish	4	1.6
Legumes: lentils or beans or *garbanzos*	4	1.9
Vegetable casserole or vegetable stew	4	1.9
*Porotos con riendas*: boiled cranberry beans with spaghetti, sausage, diced pumpkin, dried chili condiment, and onions	4	0.8
*Cazuelas*: boiled beef or chicken with chopped onions and carrot; and then added to the shallow serving dish is a piece of squash, potato, and sweet corn	4	1.0
Fish: fish ceviche or baked fish	4	0.6
Fish soup	4	0.0
*Charquican*: a warm dish with ground beef, potatoes, pumpkin, white corn, onion, peas and corn; and often topped with a fried egg	4	0.9
*Tortilla* de verduras: eggs with vegetables (*frittata* style)	4	1.1
Vegetable soup	4	1.8
Leftovers for lunch	4	3.4
*Empanadas*: any kind	4	0.5
Shredded beef	4	0.3
*Pantrucas*: Chilean dumpling stew	4	0.3
Baked chicken	4	0.8
*Cochayuyo* (algae) salad	3	0.4
La Araucanía Region		
Fruit	5	5.5
Salads	5	6.2
*Cazuelas*: boiled beef or chicken with chopped onions and carrot; and then added to the shallow serving dish is a piece of squash, potato, and sweet corn	5	1.4
*Diguenes* (*Cyttaria espinosae*) salad: an orange-white colored edible fungus typically consumed as a salad	5	1.6
*Pastel de choclo*: a sweetcorn pudding in a shallow dish in which minced beef cooked with onions, paprika, other spices, and sometimes chicken is placed in the bottom of the dish with slices of hardboiled egg, olives and raisins	5	0.6
*Piñones*: edible pine seeds from the Chilean tree species *Araucaria araucana*	5	0.8
Tea	4	5.7
Coffee	4	4.9
Mate	4	3.4
Avocado	4	1.8
Scrambled eggs	4	3.0
Cheese	4	1.9
*Porotos con riendas*: boiled cranberry beans with spaghetti, sausage, diced pumpkin, dried chili condiment, and onions	4	0.7
Chilean salad: tomatoes, onions, cilantro	4	1.1
Legumes: lentils	4	0.8
Seafood or mussel soup	4	0.5
*Carbonada*: beef stew	4	0.9
Cooked fava beans	4	1.5
Leftovers for lunch	4	3.1
Homemade bread	4	3.0
*Tortilla al rescoldo*	4	0.7
*Pantrucas*: Chilean dumpling stew	4	0.2
*Cochayuyo* (seaweed) salad	4	0.4
*Pollo arvejado*: chicken with peas	4	0.6
*Humitas*: mashed fresh sweet corn, onion, basil, and butter or lard wrapped in corn husks and baked or boiled	4	0.8
Cooked sweetcorn	4	1.5
*Kuchen*: pie-like pastry, with a thick, “cakey” crust and a sweet custard based or chopped fruit topping	4	0.5
Homemade marmalade	4	4.0
Legumes: *garbanzos* and/or peas	3	0.5

### Regional sustainability scores of traditional culinary preparations

When perceptions of the five sustainable diet dimensions were averaged across participants in each region, at least 60% of participants identified nearly all culinary preparations as sustainable (Table 4). The culinary preparations perceived as the most sustainable were similar across regions and included salads, eggs, legumes, and fruits. In both regions, many participants described eggs as relatively inexpensive. Participants were less likely to associated eggs either positively or negatively with environmental impact. Some highlighted that eggshells are compostable, while others expressed concerns about large-scale egg producers in which hens are overexploited and, thus, “*invasive for both the ecosystem and humans. It’s not like the happy hen’s egg, is it?*”. Participants in the Metropolitan Area mentioned different strategies that they perceived as supporting the sustainable consumption of legumes throughout the year. One participant explained: “*At the beginning of the legume season, I have someone that I pay who brings me enough kilograms of beans and lentils for the year*”. Another participant noted: “*We freeze beans and use them throughout the year*”. In the Metropolitan Area, the culinary preparations perceived as least sustainable were also among those considered least economically accessible (Tables 3 and 4), including fish, fish soup, shredded beef, and empanadas. In the La Araucanía Region, seafood soup and *piñones* were also perceived as among the least sustainable culinary preparations (Table 4). Few participants explained why seafood soup was viewed as physically or economically inaccessible in either region. However, participants in the La Araucanía Region reported that *piñones* are difficult to obtain due to their short harvest season and declining productivity of Araucaria trees - both factors that have made piñones increasingly expensive.

**Table 4:** Overall sustainability scores of the traditional culinary preparations.

Culinary preparation	Average sustainability score (%)
Metropolitan Region	
Fruit: plain or fruit salad	91
Salads: Chilean salad, or tomato, or mixed salad	90
Eggs with tomato or onion	82
Tea/Coffee/Mate	81
Vegetable soup	78
Legumes: lentils or beans or *garbanzos*	78
*Tortilla* de verduras: eggs with vegetables (frittata style)	76
*Porotos granados* or *porotos con mazamorra*: a traditional Chilean stew of mainly cranberry beans, corn and squash	75
Vegetable casserole or vegetable stew	72
*Cochayuyo* (algae) salad	72
Whole wheat bread with avocado or avocado with onion	72
*Charquican*: a warm dish with ground beef, potatoes, pumpkin, white corn, onion, peas and corn; and often topped with a fried egg	70
Cheese with tomato	69
*Causeo*: tomato mixed with tuna fish	69
*Cazuelas*: boiled beef or chicken with chopped onions and carrot; and then added to the shallow serving dish is a piece of squash, potato, and sweet corn	67
*Porotos con riendas*: boiled cranberry beans with spaghetti, sausage, diced pumpkin, dried chili condiment, and onions	64
Leftovers for lunch	61
Baked chicken	61
*Ulpo*: toasted wheat flour with or without milk	60
*Pantrucas*: Chilean dumpling stew	58
Fish: fish ceviche or baked fish	55
Fish soup	52
Shredded beef	48
*Empanadas*: any kind	39
La Araucanía Region	
Salads	93
Scrambled eggs	93
Homemade bread/*Tortilla al rescoldo*	89
Tea/Coffee	88
Chilean salad: tomatoes, onions, cilantro	88
*Porotos con riendas*: boiled cranberry beans with spaghetti, sausage, diced pumpkin, dried chili condiment, and onions	87
Legumes: lentils	80
Fruit	85
*Cazuelas*: boiled beef or chicken with chopped onions and carrot; and then added to the shallow serving dish is a piece of squash, potato, and sweet corn	85
Legumes: *garbanzos* and/or peas	80
*Carbonada*: beef stew	80
*Cochayuyo* (algae) salad or with potatoes	78
*Pollo arvejado*: chicken with peas	77
Leftovers for lunch	77
Mate	75
Cooked fava beans	75
Cheese	74
Avocado	73
*Pantrucas*: Chilean dumpling stew	71
Cooked sweetcorn	71
*Humitas*: mashed fresh sweet corn, onion, basil, and butter or lard wrapped in corn husks and baked or boiled	69
*Kuchen*: pie-like pastry, with a thick, “cakey” crust and a sweet custard based or chopped fruit topping	66
Homemade marmalade	66
*Pastel de choclo*: a sweetcorn pudding in a shallow dish in which minced beef cooked with onions, paprika, other spices, and sometimes chicken is placed in the bottom of the dish with slices of hardboiled egg, olives and raisins.	65
*Diguenes* (*Cyttaria espinosae*) salad: an orange-white colored edible fungus typically consumed as a salad	63
Seafood or mussel soup	61
*Piñones*: edible pine seeds from the Chilean tree species *Araucaria araucana*	55

## Discussion

In both the Metropolitan and La Araucanía regions, nearly all culinary preparations were perceived as easy to obtain and nutritionally adequate. Although some regional differences emerged in economic accessibility, avocado was considered economically inaccessible in both regions. The greatest regional contrast was observed in perceptions of environmental impact: while participants in the Metropolitan Region expressed mixed views, most participants in the La Araucanía Region perceived all preparations as environmentally positive. In both regions, fried empanadas and sugary cakes/jams were identified as the least healthy options. Overall, most preparations received an average sustainability score above 60%, with salads, fruits, and eggs ranking highest. Participants generally reported liking nearly all preparations, although many were consumed less than once per week. One notable exception was leftovers, which were consumed for lunch about three times per week in both regions. More than two-thirds of participants in both regions rated the traditional preparations as sustainable across all five dimensions. Only one preparation (*piñones*) fell below this threshold in the La Araucanía Region, compared with five in the Metropolitan Area. This difference likely reflects the strong tradition of using local and seasonal foods in the La Araucanía Region ^32^. This interpretation is further supported by participants’ greater awareness of sustainability-related issues and their consistent consumption of seasonal foods. In contrast, participants in the Metropolitan Region reported consuming traditional foods primarily based on availability, including items transported from other regions.

When interpreting regional differences in the findings, it is important to consider the geographical context and its relationship with sociocultural determinants of food choice. The La Araucanía Region has a larger proportion of rural areas and a greater variety of climates and productive ecosystems compared to the Metropolitan Area, including mountains, valleys, coastal areas, and other microclimates. These geographical characteristics are reflected in our findings. Unlike participants in the Metropolitan Area, those in the La Araucanía Region reported greater consumption of traditional culinary preparations and generally perceived these preparations as being closely linked to the environment in positive ways. These findings suggest that geographical diversity plays an important role in shaping population-level culinary traditions associated with specific places ^33^ ^,^ ^34^.

In both regions, participants reported strong preferences for traditional foods, reflecting the well-established role of taste and place are important determinants of food choice ^35^. The gap between positive taste preferences and low weekly consumption may reflect the influence of multiple, interconnected sustainability dimensions on food choices. For example, in the Metropolitan Area, some of the culinary preparations perceived as least economically accessible (e.g., shredded beef, cazuelas, empanadas, baked chicken, and fish) were also among those least likely to be perceived as environmentally positive because they contained “meat and/or fish”. In the La Araucanía Region, the preparations least often perceived as environmentally positive were predominantly animal-based dishes, including cazuelas, beef stew, chicken, and seafood soup, reflecting the common perceptions that meat and seafood production harm the environment ^36^. Notably, participants found it difficult to assess environmental impact. In the Metropolitan Area, many conflated household-level issues (e.g., smoke generated during frying) with broader environmental degradation. Another misconception across both regions was the belief that avocado has a positive environmental impact, despite its high blue-water footprint (i.e., non-rainwater) and the pressure its production places on drought-affected areas of Chile ^37^ ^,^ ^38^ ^,^ ^39^. A distinction exists between environmental perception - the subjective assessment of environmental impacts - and measured environmental impacts derived from objective methods such as life cycle assessment. While objective metrics provide quantifiable estimates of environmental impacts associated with diet, our subjective methods and results elucidate population-level knowledge gaps regarding the relationships between diet and climate change. Addressing these gaps may be important for the design of future interventions aimed at promoting greater consumption of sustainable diets.

Another notable regional difference was that participants in the Metropolitan Region perceived meat- and seafood-based traditional dishes as among the least sustainable, a pattern that was not observed in the La Araucanía Region. The negative environmental implications of animal-based foods are well documented ^40^. However, this regional difference in sustainability perception may also be related to other contextual factors ^41^. When discussing all traditional culinary preparations of this study, including animal- and sea-based foods, as having a positive environmental impact, those in the La Araucanía Region stated “*But are they natural foods, aren’t they? Because I don’t see any, what I call, junk food here*” and “*I find that none* [damage the environment] *because they all come from... from nature!*”. In contrast, some participants in the Metropolitan Area associated the environmental dimension of sustainable diets with household-level air pollution generated during cooking, such as “*Hey, when you fry* [foods]*, that oil burns and the smoke could affect the environment*”.

In the Metropolitan Area, residents have more limited access to locally produced foods via through local vendors, home gardens, and food preservation practices, which reduces access to foods that are both sustainable and economically accessible ^42^. As a result, fish- and seafood-based dishes were perceived as among the most difficult preparations to obtain, both physically and economically. Overall, traditional preparations were considered more expensive in the Metropolitan Area, likely reflecting fewer opportunities for home food production and stronger reliance on the commercial food industry. Conversely, the more rural La Araucanía Region maintains stronger rural-urban connections that facilitate access to local foods. One possible explanation for the greater availability of local foods in the La Araucanía Region is the close relationship between regional food traditions and Indigenous communities. According to the 2024 census ^43^, the La Araucanía Region has the highest proportion of Indigenous individuals, primarily Mapuche, in Chile. For the Mapuche people, food preparation is closely linked to cultural identity and everyday life. This perspective was reflected in one participant’s statement: “*I don’t like buying things* [foods] *that I don’t see how they’re made*”.

This study was limited to the 25-27 culinary preparations assessed in each region. Additional limitations are associated with the use of a semi-quantitative food frequency questionnaire, especially for assessing neglected and underutilized species ^44^. Most notably, the questionnaire was subject to recall bias and was designed to assess usual intake of the culinary preparations included in the study rather than overall dietary intake. We also did not assess weekly dietary diversity or the number of days per week participants consumed each culinary preparation. Thus, weekly consumption of traditional preparations may have been underestimated among participants who regularly rotate multiple traditional dishes but consume any given preparation only once or twice per month. However, a strong taste preference for a culinary preparation does not necessarily indicate a willingness to consume it more than once per week. There is also a conceptual distinction between individual foods (e.g., avocado) and composite culinary preparations (e.g., *cazuela*), which may affect perceptions of physical and economic accessibility. Furthermore, participants may have responded differently depending on the interviewer. While we tried to standardize the use of the semi-structured interview guide through training, each of the three research assistants may have elicited different responses. Consequently, it is impossible to determine whether interviewer style influenced the responses, particularly in relation to cross-cultural comparisons. The predominance of homemakers in the study sample reflects both the greater participation of women and a potential source of bias, given that homemakers are generally highly familiar with food purchasing and preparation. The gendered division of labor remains prevalent in Latin America, including Chile, where women predominantly assume caregiving and domestic responsibilities - a pattern that was further exacerbated during crises such as the COVID-19 pandemic ^45^. This constitutes a strength in capturing situated experience regarding food selection and preparation, but it can also be a limitation, since this type of expertise and knowledge is not necessarily attributable to the general population. Although our sample was not nationally or regionally representative, the findings provide important updates beyond previous studies that relied on nationally representative food consumption data collected in 2010. Those studies excluded traditional culinary preparations and may no longer reflect current dietary patterns ^46^ ^,^ ^47^.

Climate-related awareness of dietary choices varies across countries, and limited research has explored public perceptions of the relationship between diet and climate change, especially in Chile. As climate change intensifies, population-level diets have an even greater role in both mitigating environmental impacts and avoiding further contributions to climate change. As evidence has accumulated regarding the numerous negative impacts the industrialized global food system has on the acceleration of climate change, so have food-based dietary guidelines that consider the environmental dimension of sustainability; including Chile’s new food-based dietary guidelines launched in December 2022. The Chilean food-based dietary guidelines and their ten key recommendations are organized around six overarching principles: adoption of sustainable diets; strengthening of sustainable food systems; prioritization of fresh, natural, and minimally processed foods; protection of Chile’s biodiversity; appreciation of homemade culinary preparations; and respect for food cultures ^7^. The social perceptions converge with these recommendations; in particular, participants expressed a high appreciation for homemade culinary preparations and reported favorable taste preferences for many fresh, natural, and minimally processed foods that are emblematic of Chilean food culture and regional diversity. However, social perceptions observed in this study diverged from these principles, as many participants did not prioritize several fresh, naturally, and minimally processed foods regarding traditional Chilean dishes in their usual intake. Additionally, participants in both regions demonstrated limited awareness of how diet can negatively or positively affect Chile’s biodiversity. Taken together, these results may help support the operationalization of Chile’s food-based dietary guidelines by informing interventions designed to increase public awareness of the relationships between climate and diet, as well as in sharing efficient ways to prepare traditional Chilean preparations, such as quick soaking legumes.

Future studies should include surveys assessing knowledge, attitudes, and practices related to sustainable diets promoted in food-based dietary guidelines and other national policies. For example, such surveys could measure actual weekly consumption of traditional culinary preparations recommended in the food-based dietary guidelines, as well as barriers to following these guidelines. These data would help identify obstacles to policy implementation and inform the design of more effective interventions.

## Conclusions

This study provides a methodological approach for understanding how sustainable diets can be translated into context-specific practice. Identifying the most and least sustainable traditional culinary preparations across distinct dimensions of sustainable diets generates important information for implementing food-based dietary guideline that includes sustainability, such as those adopted in Chile. Strategically promoting different traditional culinary preparations in varied contexts may encourage their consumption and raise awareness of the diet-climate change connection. Ultimately, the successful adoption of sustainable diets depends on the capacity of local food systems to meet this demand, give the close interdependence between sustainable diets and local food systems.

## Data Availability

The research data are available upon request to the corresponding author.
